# The Effects of Seed Inoculation with Bacterial Biofilm on the Growth and Elemental Composition of Tomato (*Solanum lycopersicum* L.) Cultivated on a Zinc-Contaminated Substrate

**DOI:** 10.3390/microorganisms12112237

**Published:** 2024-11-05

**Authors:** Mirta Esther Galelli, Josefina Ana Eva Cristóbal-Miguez, Eliana Cárdenas-Aguiar, Ana Rosa García, Antonio Paz-González, Gabriela Cristina Sarti

**Affiliations:** 1 Agrofood Area, Department of Applied Biology and Food, Faculty of Agronomy, University of Buenos Aires, Av. San Martín 4453, Buenos Aires C1417DSE, Argentina; mgalelli@agro.uba.ar; 2 Inorganic and Analytic Chemistry Cathedra, Department of Natural Resources and Environment, Faculty of Agronomy, University of Buenos Aires, Av. San Martín 4453, Buenos Aires C1417DSE, Argentina; amiguez@agro.uba.ar (J.A.E.C.-M.); agarcia@agro.uba.ar (A.R.G.); or g.sarti@udc.es (G.C.S.); 3 AQUATERRA Reseach Group, Interdisciplinary Center for Chemistry and Biology, CICA, University of A Coruna, As Carballeiras, s/n Campus de Elviña, 15008 Coruna, Spain; eliana.cardenas@col.udc.es

**Keywords:** *Bacillus subtilis*, biofilm inoculation, zinc excess, toxicity alleviation, urban agriculture

## Abstract

Biofilm obtained from *Bacillus subtilis* subsp. *spizizenii* inoculated on vegetable seeds has been shown to have plant growth-promoting capacity. Seed inoculation with biofilm produced by this strain could also reduce the adverse effects on plant growth caused by soil or substrate heavy metal overabundance. Therefore, the objective of this work was to evaluate the impact of biofilm inoculated on tomato (*Solanum lycopersicum* L.) seeds, which were planted on a substrate with artificially added zinc. First, seeds of the Río Grande tomato variety were exposed to increasing zinc concentrations, namely: 50, 100, 200, and 400 ppm, with and without bacterial biofilm inoculation. Zinc addition and seed inoculation affected germination parameters. For example, an extra 200 and 400 ppm of zinc led to high toxicity. Biofilm inoculation, however, reduced the noxious effects of excess zinc, bringing acute toxicity down to moderate. Then, tomato plants growing from inoculated and non-inoculated seeds were cropped for 4 months in both substrates with 400 ppm zinc and without added zinc. Extra zinc addition significantly (*p* < 0.05) reduced tomato root and shoot biomass, plant height, and fruit number at harvest time. However, seed biofilm inoculation avoided the harmful effect of zinc on plant growth parameters, fruit yield, and fruit quality. The roots and shoots of plants growing on contaminated substrates showed very noticeable increases in zinc levels compared to the control, while fruits only showed a much weaker zinc gain, even if this was significant (*p* < 0.05). Moreover, root shoot and fruit concentrations of elements other than zinc, (nitrogen, phosphorus, potassium, calcium, magnesium, sulfur, iron, manganese, copper, lead, and cadmium) were not or only weakly affected by the addition of this metal to the substrate. In summary, the biofilm of *B. subtilis* proved to be effective as a bioinoculant to alleviate negative effects on tomatoes cropped in a substrate with excess zinc.

## 1. Introduction

Anthropogenic activities have increased Zn contamination in the environment, which is particularly reflected in the accumulation of this non-biodegradable heavy metal in agro-food products. To cope with this environmental problem there is a need to develop integrated crop management strategies. Zn is highly mobile in soil and highly bioavailable to plants, easily reaching phytotoxic concentrations [1]. Noxious levels of Zn in edible vegetables are promoted when arable land is contaminated by agricultural activities, largely as a result of the application of phosphate fertilizers, pesticides, manure, etc. In addition, Zn release to the environment originates from other anthropogenic activities, mainly mining, smelting, and burning of fossil fuels [2,3,4,5,6]. As a heavy metal, Zn in the soil can be found in different chemical forms, principally in solution and weakly or strongly bound to soil components, depending on several factors, such as soil type, climate, and plant species [2,7,8,9]. The soluble Zn form has been found to be the main source of plant uptake [7,9].

Zn is an essential micronutrient for living organisms, second only to Fe. It has fundamental catalytic and/or structural functions, being crucial for the activity of more than 300 enzymes. On the other hand, in excessive amounts, it is toxic to living organisms, directly and indirectly affecting ecosystems, for example, by altering the diversity of soil micro-organisms [9,10]. Therefore, Zn concentrations in agricultural soils may range from very low to excessive, which may trigger either plant deficiency or phytotoxicity, respectively.

The toxic effects of Zn can be associated with enzyme inhibition, the generation of oxygen free radicals, and the alteration of membrane integrity and permeability, among others [4,9]. In addition to direct toxic effects, indirect effects of the metal arise from limitations of water and mineral acquisition, affecting crop development [8]. Living organisms bolster up evolved systems to survive in metal-contaminated soils, which result from the activation of adaptive mechanisms that prevent the metal from reaching the target sites of toxicity. Examples of this include the production of substances capable of retaining Zn, metal sequestration in vacuoles, a reduction in metal absorption, and the activation of antioxidants [4].

The negative effects of Zn on crops can be reduced through the use of plant growth-promoting bacteria (PGPBs). Some of the mechanisms involved are related to the synthesis of indole acetic acid-type growth regulators and cytokines, nitrogen fixation, the prevention of excessive ethylene secretion, the production of siderophores, and antibiotic substances [6,11]. These micro-organisms have a very varied metabolism that is highly dependent on the habitat in which they are found. Thus, some PGPBs are capable of solubilizing heavy elements from mineral compounds when there is little bioavailable metal, increasing their uptake. Other PGPBs possess the ability to synthesize substances that immobilize heavy metals when they are in excess, thus reducing their bioavailability. For example, they can produce exopolysaccharides, which are considered a system for protecting ecosystems against heavy metal pollution [6,9,12,13]. An important aspect to consider about these bacteria is that their ability to promote plant growth depends not only on the bacteria used itself but also on the crop to which they are applied. Therefore, the same bacteria can perform very well for one crop but not for a different one [3,11,14].

Bacteria of the genus *Bacillus* are among the most researched PGPB, as they are predominant in soil and rhizosphere, accounting for more than 95% of Gram-positive bacteria. They are also among the most widespread endophytic bacteria [3]. An important characteristic of this genus lies in its ability to form endospores. These are resistance structures that allow cells to maintain their viability under adverse environmental conditions. This is a fact of importance when considering members of the genus *Bacillus* for the formulation of bioinoculants for commercial use, which, when stored for long periods, must retain their viability [3]. In addition, some strains have the ability to absorb metals and can grow in contaminated soils [9,15].

In particular, *Bacillus subtilis* subsp. *spizizenii* has been shown to be efficient in promoting the growth of horticultural species, such as *Lactuca sativa* and *Solanum lypopersicum*, and legumes, like *Glycine max*, producing phytohormones, antifungal substances and solubilizing phosphates [16,17,18,19]. An important aspect linked to the performance of this bacterium lies in its ability to produce biofilm, which protects bacterial cells from adverse environmental conditions. Biofilm is characterized by a three-dimensional structure in which different types of cells coexist, including the planktonic or free *Bacillus* form and the spores, immersed in a matrix of exopolysaccharides, as well as minor amounts of proteins, DNA, waste substances, etc. In a previous work, it has been shown that the biofilm of *Bacillus subtilis* subsp. *spizizenii* promotes tomato growth better than the corresponding planktonic form [19]. Moreover, the presence of spores from the biofilm produced by this bacterium has been verified [18], which would advocate for its use as a bioinoculant.

Tomato, belonging to the solanaceae family, is one of the most cultivated vegetables in the world, with a production close to that of the potato [20]. Indeed, it represents the most valuable garden crops, in terms of food. Tomatoes have beneficial effects on human health, mainly due to their high content of antioxidants (lycopene, ascorbic acid, α-tocopherol, phenolic compounds, among others), folic acid, flavonoids, and minerals such as potassium and calcium [21,22]. Currently, tomato production is facing numerous biotic and abiotic challenges [23]. In particular, the presence of heavy metals reduces the nutritional value of tomatoes [24,25].

Seed inoculation with biofilm produced by *B. subtilis* subsp. *spizizenii* has been shown to promote plant growth and yield and to foster other beneficial effects on vegetables such as lettuce [17,18]. The objectives of this work were: (1) to determine whether inoculation of tomato (*Solanum lycopersicium* L.) seeds with *B. subtilis* biofilm produces a growth-promoting effect, reducing noxious effects caused by plant cultivation on a substrate with high levels of Zn and (2) to assess how both inoculation and a high substrate Zn level impact on the elemental composition of shoot, root, and fruits of the studied crop.

## 2. Materials and Methods

### 2.1. Bacterial Cultivation to Obtain Planktonic Cells and Biofilm

*Bacillus subtilis* subsp. *spizizenii* used in this study was originally acquired from the American Type Culture Collection (ATCC), Accession No. 6633. It was stored at the campus of the Faculty of Agronomy, Buenos Aires University (FAUBA). Before being used, the bacterium strain was initiated from the stock culture in nutritive agar media (meat extract, 3 g; peptone, 5 g, agar, 25 g and deionized H_2_O up to 1 L) in a bacterial incubation chamber at 30 °C for 24 h. Subsequently, planktonic cells and biofilm from *B. subtilis* were obtained using identical culture media. This was a liquid Minimal Salt Medium (MSM) with 1% glycerol and 35 mM L-glutamic acid. The MSM contained 1 g/L K_2_HPO_4_; 0.3 g/L KH_2_PO_4_; 0.5 g/L NH_4_Cl; 0.1 g/L NH_4_NO_3_; 0.1 g/L Na_2_SO_4_; 0.01 g/L MgSO_4_ 7H_2_O; 1 mg/L MnSO_4_ 4H_2_O; 1 mg/L FeSO_4_7H_2_O; 0.5 g/L CaCl_2_; and 0.01 g/L EDTA in deionized water at pH = 7 [18,26].

To produce planktonic cells, the bacterium was cultivated at 30 °C, under agitation at 150 rpm. Then, to develop biofilm at the air–liquid interface, cultivation was carried out under static conditions. Incubation was performed in 500 mL Erlenmeyer’s, adding 150 mL of MSM with 1% glycerol, as a carbon source, and 35 mM L^−1^ glutamic acid. Cultivation was carried out at 30 °C and for 96 h. The biofilm developed in this culture medium was used for laboratory germination tests and greenhouse trials.

### 2.2. Performance of B. subtilis in the Presence of Zn

#### 2.2.1. Bacterial Growth in the Planktonic State

To assess the impacts of Zn level on microbial growth, *B. subtilis* was cultivated in the above-mentioned liquid medium with increasing concentrations of Zn, namely, 50, 100, 200, and 400 ppm. The metal was added to the medium as zinc chloride (ZnCl_2_). Cultivation in the planktonic state was performed for 96 h, at 30 °C, in a rotatory agitation incubator working at 150 rpm. Under these conditions, turbidity was developed in the bacteria growth media. Growth curves were assessed spectrophotometrically by measuring absorbance at 610 nm (OD_610nm_). To construct these curves, 8 successive absorbance measurements were collected between 0 and 96 h. Three repetitions per measurement were evaluated for all the Zn levels studied.

#### 2.2.2. Quantification of Biofilm Production

Biofilm was produced under different conditions, including a control treatment and treatments with 50 to 400 ppm Zn. Incubations were carried out using the methodology above described in Section 2.1. Biofilm was extracted from the Erlenmeyer flask manually, using a glass rod. Then, it was dried to constant weight on filter paper at 40 °C quantification. Figures S1 and S2—presented in Supplementary Materials—illustrate the biofilm obtained for the air–liquid interface and biofilm after removing the liquid medium, respectively.

### 2.3. Experimental Site and Plant Material

The experimental work was carried out at the laboratory and greenhouse facilities belonging to the Faculty of Agronomy, University of Buenos Aires (FAUBA) with geographical coordinates 34°45′ S latitude, 60°31′ W longitude. First, germination tests in the laboratory were set up, and then greenhouse pot experiments were set up. In the laboratory, the germination and root length percent were determined; meanwhile, under greenhouse conditions, plant growth, fruit production, and elemental composition were quantified.

Both laboratory and greenhouse experiments have been conducted using the seeds of the Rio Grande variety of *S. lycopersicum*. This is a determinate tomato variety, commercially produced in Argentina. This variety is characterized by a relatively large growth cycle so that flowering and ripening are reached later than in other varieties.

### 2.4. Seed Germination Tests

To analyze the impact of increasing Zn concentration and biofilm inoculation on tomato seed germination, five different levels of Zn, namely, 0, 50, 100, 200, and 400 ppm have been used. Each Zn dose was tested with and without inoculation, totalizing ten different treatments. Germination tests to assess the impact of increasing Zn concentrations and seed inoculation of the tomato Río Grande variety will be next outlined. Previously, the seeds were disinfected with 70% alcohol and washed three times with sterile distilled water to remove epiphytic microorganisms. Inoculated treatments were obtained by thoroughly mixing tomato seeds and biofilm, as, in this way, the biofilm remained attached to the seeds due to its great adhesiveness.

The germination tests were carried out in sterile Petri dishes, which were prepared with a layer of sterile cotton wool covered with filter paper of pore size equivalent to Whatman^®^ No. 3 paper. The filter paper was moistened with different solutions according to the test: with 5 mL of sterile distilled water (control) or Zn solutions with 50, 100, 200, and 400 ppm. In all the ten treatments studied, 30 seeds were used and kept in darkness at 22 °C for 10 days. The criterion for germination was the visible appearance of a 2 mm long radicle [27]. For each treatment, the relative germination percentage (RG) was calculated according to:

(1) $$RG\;\left(\%\right)=\left(\frac{number\;of\;germinated\;seeds\;in\;a\;treatment}{number\;of\;germinated\;seed\;in\;control\;treatment}\right)\times 100$$

Next, the seedlings continued to grow under daylight conditions for 5 days. Then 15 days, after the beginning of the experiment, seedling radicles were measured. Relative root elongation (RRE) and relative percentage germination index (GI) were calculated for each treatment as follows:

(2) $$RRE\;\left(\%\right)=\left(\frac{radicle\;length\;in\;a\;treatment}{radicle\;length\;in\;control\;treatment}\right)\;\times \;100$$

(3) $$GI\;\left(\%\right)=\left(\frac{RG\;\times \;RRE}{100\;}\right)\;$$

GI is commonly used as a toxicity criterion that allows one to interpret how toxic the presence of Zn in the substrate is for the seed. This index encompasses the three main successive phases of germination, namely, imbibition, emergence, and radicle elongation, classifying the effect as toxic, moderate toxicity, or high toxicity.

To evaluate the results, relative toxicity criteria were considered [28,29]. Therefore, impacts have been classified as non-toxic, moderately toxic, or highly toxic depending on the relative germination index (Table 1).

### 2.5. Greenhouse Experiments

Experiments under greenhouse conditions were carried out to analyze extra substrate Zn and inoculation impacts on plant growth, fruit production, and elemental composition in different parts of the tomato plant. These experiments were conducted under natural light. The following treatments have been established: (1) control, without inoculation and Zn surplus; (2) I, inoculated seeds growing without Zn surplus; (3) Zn, non-inoculated seeds plus substrate with 400 ppm Zn surplus; (4) I + Zn, seed inoculation plus substrate with 400 ppm Zn surplus.

#### 2.5.1. Preparation of Substrate with Excess Zn

The substrate used to cultivate tomatoes in the greenhouse trials was a mixture of commercial substrate and compost in a 3:1 ratio. The commercial substrate had a pH of 5.8, 50% moisture content, 55% organic matter content, 45% ash, and a carbon/nitrogen ratio of 30%. As a heavy metal, Zn could react over time with substrate components, resulting in its retention as a non-bioavailable fraction. Therefore, the level of total metal required to maintain a concentration of 400 ppm bioavailable Zn in the substrate had to be evaluated.

The zinc concentration added to the substrate was 400 ppm. This figure was based on the results obtained in the seed toxicity bioassays (Section 3.3), which will be described later. Secondarily, permissible limits of heavy metals in soils may differ widely depending on the regulations of different counties. For example, the Dutch Soil Quality Standards consider 140 ppm Zn as a representative average limit for soils [30]. However, in Argentina, the upper limit for a Zn non-contaminated soil has been established at 300 ppm, but Zn soil concentrations up to 600 ppm are allowed for food production [31]. Therefore, in practice, adding 400 ppm Zn to the experimental substrate would allow us to evaluate the biofilm effect on alleviating Zn toxicity in tomato plants.

To assess metal availability, different concentrations of Zn were added to the substrate and allowed to stabilize for one month. After that, bioavailable Zn was measured with the aid of the chelating agent diethylenetriaminepentaacetic acid (DTPA) [32]. For this purpose, 10 g of substrate was placed in 20 mL of DTPA-CaCl_2_-TEA (0.005 M DTPA, 0.01 M CaCl_2_, and 0.1 M triethanolamine (TEA)) extracting solution, which was adjusted to pH 7.3. Next, after 2 h of stirring, the Zn concentration was measured using atomic absorption spectrophotometry. Following the above procedure, no retention of Zn by the mixture of material employed as substrate was observed. This meant that the total metal added as ZnCl_2_ was retrieved as bioavailable at a concentration of 400. The ZnCl_2_ salt metal was homogeneously mixed with the substrate and left to stabilize for one month. Then, this substrate with extra Zn was used for tomato cultivation in the treatments Zn and I + Zn.

#### 2.5.2. Inoculation, Sowing and Growth Conditions

First, tomato seeds have been inoculated with biofilm. The inoculation was conducted separately for the treatments I and I + Zn seeds, while control and Zn treatments did not receive biofilm. Then, 25 seeds per treatment were sown in germination trays with cells of approximately 5 cm diameter and 7.5 cm depth (0.15 L), which were filled with the substrate described above, with or without excess Zn, in accordance with the treatment. After 30 days, the seedlings were transplanted into 7 L pots with the corresponding substrate, and tomato plants were allowed to grow for 4 months until harvest time. Experiments have been conducted during the austral spring and summer, from October to April. Greenhouse temperature was, on average, 30 ± 5 °C. The tomato plants were irrigated two/three times per week to maintain substrate moisture near field capacity.

#### 2.5.3. Plant Growth and Fruit Production Parameters at Harvest Time

At harvest time, the weight of roots and shoots was determined after drying at 70 °C to determine the constant weight. Plant height and leaf area (LA) were also measured. In addition, tomato fruits were counted and weighed in the different treatments. Moreover, fruit quality was evaluated in the fruit juice from total soluble solids (Brix) determinations, which were carried out using a handheld refractometer.

To evaluate LA, measurements were made for each of the 25 plants per treatment. For each plant, 10 entire and fresh leaves were sampled from branches of the canopy middle part. The selected leaves were of a medium size and no more than a leaf per branch was collected. Each leaf was put over a white background and fully expanded by covering it with a 3 mm wide glass. Then, it was photographed. The area of each leaf was estimated using the IMAGE J software 1.8.0. for scientific image analysis [33].

### 2.6. Chemical Analysis

Macroelements (C, N, H, P, K, Ca, Mg, S) and microelements (Fe, Mn, Cu, Zn, Pb, Cd) were determined at tomato root, shoot, and fruit samples of each treatment. Plants were first air-dried and then oven-dried at 70 °C, as described before, to obtain a constant weight. Then, they were ground to a fine powder using a pestle and mortar. Elemental analysis of total C, N, and H was carried out by dry combustion in a FlashEA1112 elemental analyzer equipped with a MAS200 sampler (Thermo-Finnigan, Somerset, NJ, USA). The rest of the elements were determined after mineralization using acid digestion. Total macro (P, K, Ca, Mg, S), micronutrients (Zn, Cu, Fe, Mn), and heavy metals (Pb, Cd) were quantified using inductively coupled plasma mass spectrometry (ICP-MS) in an ELEMT XR (Thermo-Finnigan, USA).

Mineralization was implemented in a microwave digestion oven (CEM model MDS-2000, CEM Corporation, Matthews, NC, USA). The standard digestion protocol US-EPA SW 846-3051 was used [34]. Ground tomato samples (500 mg) were accurately weighed and placed into a Teflon PFA digestion vessel, and 10 mL of concentrated nitric acid was added. The vessels were capped and deposited inside the microwave digestion system. At the end of the digestion, the solutions were filtered through cellulose nitrate membrane filter paper with a pore size of 0.45 µm (Milipore, Milan, Italy).

The capability of the tomato plant to accumulate a particular element in shoots or fruits with respect to roots was assessed using the so-called translocation factors (TFs). Translocation factors for shoots (TF_shoot_) and fruit (TF_fruit_) were defined as the ratio of the total element concentration in the shoot (C_shoot_) and the fruit (C_fruit_) relative to that in the roots (C_root_), respectively [25]. Elements accumulated in shoots or fruits for TFs > 1, while for TFs < 1 elements accumulated in roots. Therefore, the used TFs are given by the equations below:

(4) $${\mathrm{TF}}_{\mathrm{shoot}}=\left(\frac{Element\;concentration\;in\;the\;plant\;shoot}{Plant\;root\;element\;concentratiom}\right)\;$$

(5) $${\mathrm{TF}}_{\mathrm{fruit}}=\left(\frac{Elememt\;conccnetration\;in\;the\;fruit}{Plant\;roort\;element\;concentration}\right)$$

### 2.7. Experimental Design and Statistical Analysis

The laboratory and greenhouse experiments were conducted in a completely randomized design. Ten treatments have been examined in the germination test, using 60 seeds per treatment. Yet, in the greenhouse experiment, four treatments have been established, with ten plants per treatment. All the treatments were evaluated in three repetitions. Analysis of variance (ANOVA) was used to determine the differences of means for each of the treatments studied in the laboratory and greenhouse experiments. Statistical significance differences were measured using the Tukey test (either *p* < 0.05 or *p* < 0.10).

## 3. Results

### 3.1. Bacterial Growth in the Presence or Absence of Zinc

The efficiency of a bioinoculant to promote plant growth and production first depends on the viability of its specific microorganisms in the environment in which it will be applied. Therefore, testing the viability of the bacterium in the presence of zinc is particularly important. The growth of *B. subtilis* with increasing Zn concentrations, up to potentially toxic levels, was evaluated by measuring absorbance at 610 nm (OD_610 nm_), using spectrophotometry, at successive time intervals of 12 h and a final interval of 24 h.

**Figure 1:**
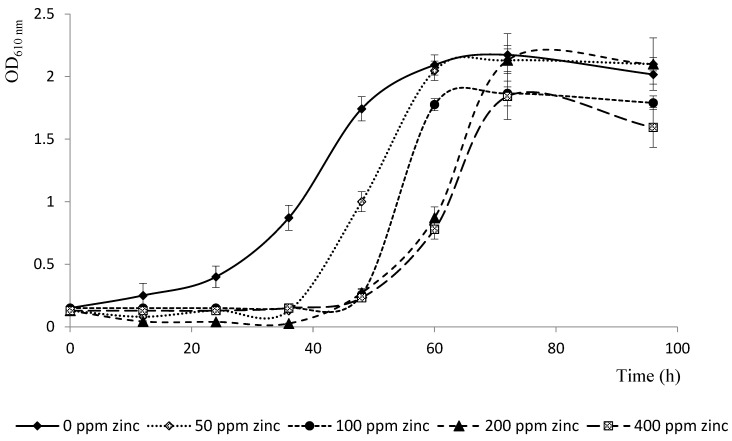
Growth of *B. subtilis* in MSM with 55 mM L-glutamic acid, 1% glycerol, without Zn (control) and with different Zn concentrations at 30 °C and 150 rpm for 96 h. Values are means ± SD (n = 3).

Figure 1 shows that the bacteria strain tested as an inoculant was able to grow on Zn-containing media at metal concentrations up to 400 ppm. However, bacterial growth in the presence of Zn showed differences compared to that of the Zn-free control. Thus, adding Zn to the growth solution resulted in longer lag phases, which were greater than 30 h for 50 to 400 ppm Zn, relative to the control. However, there were no significant differences (*p* < 0.05) in the OD_610nm_ values achieved at the stationary phase between all the studied treatments, including the control treatment. These results indicate that the bacterium is able to grow even at Zn concentrations that have been reported as toxic to plants.

### 3.2. Biofilm Production in the Presence of Zinc

**Figure 2:**
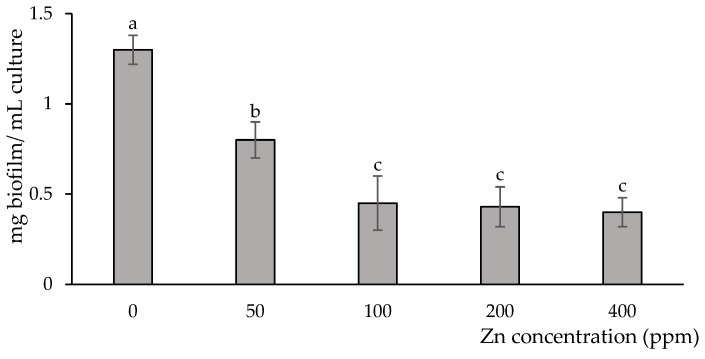
Biofilm development of *B. subtilis*. Bacteria were grown in MSM with 55 mM L-glutamic acid, 1% glycerol, with different concentrations of zinc, at 30 °C, for 96 h, under static conditions. Values are means ± SD (n = 3). Different letters represent significant differences between treatments at a probability level of *p* < 0.05.

The biofilm produced by *B. subtilis* in a liquid medium with no Zn addition showed a well-defined and structured architecture, which was built up at the Erlenmeyer air/liquid interface (Figures S1 and S2, Supplementary Materials). Without Zn, the biofilm yield was 1.35 mg biofilm/mL of culture on a dry weight basis (Figure 2). However, biofilms produced in the presence of Zn accumulated at the bottom of the Erlenmeyer flask showed more disorganized structures compared to the control treatment. Moreover, the higher the metal concentration in the liquid media, the lower the amount of biofilm produced. Thus, at 50 ppm Zn, the biofilm amount decreased by about 50% relative to the control, while at 100, 200, and 400 ppm Zn, reductions of about 70% were recorded (Figure 2).

### 3.3. Effect of Inoculation and Zinc Addition on Seed Germination

**Figure 3:**
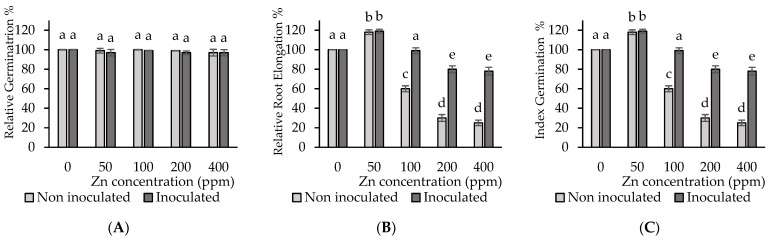
Impacts of tomato seed inoculation with *B. subtilis* biofilm for increasing Zn levels on (**A**) RG%; (**B**) RRE%; (**C**) GI%. Values are means ± SD (n = 60). Different letters indicate significant differences between treatments at a probability level of *p* < 0.05.

As before stated, the impact of Zn surplus on tomato seed development was assessed by considering relative germination (RG), and relative root elongation (RRE), both measured as a percentage, as well as the relative germination index (GI). The presence of Zn in the germination solution showed no significant (*p* < 0.05) impact on RG percentage with respect to the control treatment of both inoculated and non-inoculated seeds (Figure 3A). In contrast, RRE percentage showed major significant variations between control treatment and treatments with different Zn concentrations. Therefore, at 50 ppm Zn, RRE values were 18% higher than those of the control treatment, and this is both with and without seed inoculation (Figure 3B). However, at Zn concentrations higher than 50 ppm, significant RRE differences were observed between inoculated and non-inoculated treatments. Thus, with 100 ppm Zn in the germination solution, the RRE of non-inoculated seedlings decreased by 40%, while with 200 and 400 ppm, it decreased by 60% and 65%, respectively. Seed inoculation at 100 ppm and higher Zn concentrations showed beneficial impacts on RRE. Therefore, biofilm application avoided toxic impacts at 100 ppm Zn, resulting in RRE values similar to those of the control without Zn. Biofilm seed inoculation also reduced the noxious impacts of Zn at 200 and 400 ppm Zn by about 20% (Figure 3B).

Harmful and beneficial Zn impacts, depending on the metal concentration, were also reflected in the germination index. In fact, RRE and GI results showed a close similarity (Figure 3B,C). Again, at 50 ppm Zn, GI values were significantly higher (*p* < 0.05) than those of the control treatment, so germination exhibited a safe response to this relatively low Zn concentration (Figure 3C). However, in the non-inoculated treatment with 100 ppm Zn, GI was 60% lower compared with the control treatment, which is considered moderate toxicity. Moreover, at 200 and 400 ppm, Zn GI values were about 30% and 25% lower relative to the control treatment, respectively, which corresponds to a high toxicity level.

Seed inoculation using biofilm had an alleviating impact against Zn toxicity during germination. Thus, the treatment with inoculated seeds and 100 ppm Zn showed no significant differences (*p* < 0.05) from the control treatment. Therefore, at this Zn level, inoculation was able to reverse the noxious effect from moderately toxic to non-toxic. Inoculation at 200 and 400 ppm Zn led to GI increases of 65% and 73%, respectively, relative to non-inoculation, so that the toxicity level recovered from high to moderate (Figure 3C). These results indicate the efficiency of the biofilm’s use as an inoculant at Zn concentrations ranging from 100 to 400 ppm. Notwithstanding, the favorable impacts at 50 ppm Zn point to the presence of the so-called hormesis effect at low Zn concentrations.

### 3.4. Effects of Inoculation and Substrate Zinc Excess on Plant Growth and Fruit Yield

#### 3.4.1. Plant Growth Evaluation

**Figure 4:**
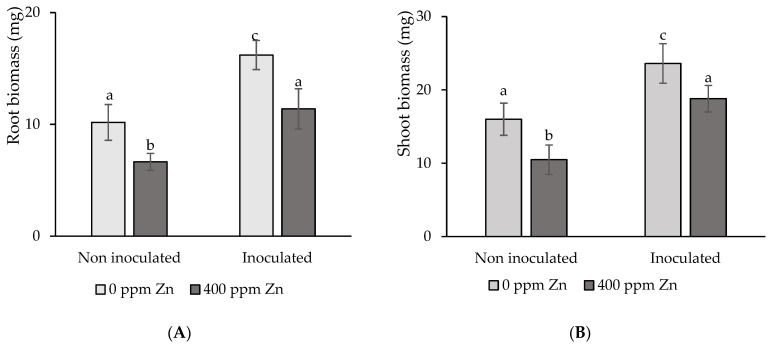
Impact of seed inoculation and 400 ppm Zn surplus on: (**A**) root and shoots (**B**) biomass of tomato plants after 4 months’ growth. Values are means ± SD (n = 10). Different letters indicate differences between treatments at the 0.05 probability level.

Adding 400 ppm Zn to the substrate had a detrimental effect on root and shoot biomass, resulting in a 47% dry mass decrease with respect to the control treatment in either case (Figure 4A,B). Seed inoculation with biofilm promoted root and shoot biomass rises of tomato plants growing both with and without Zn surplus at harvest time. Plants growing from inoculated seeds growing on a substrate without Zn (I), showed significant (*p* < 0.05) root and shoot increases in dry weights of 59% and 47%, respectively, with respect to plants growing on the control Zn-free treatment. Moreover, plants growing from inoculated seeds on a substrate with 400 ppm Zn surplus (I + Zn) showed significant (*p* < 0.05) root and shoot increases in dry weights of 71% and 79%, respectively, compared to plants growing in the non-inoculated counterpart (Zn), as shown in Figure 4A,B.

**Figure 5:**
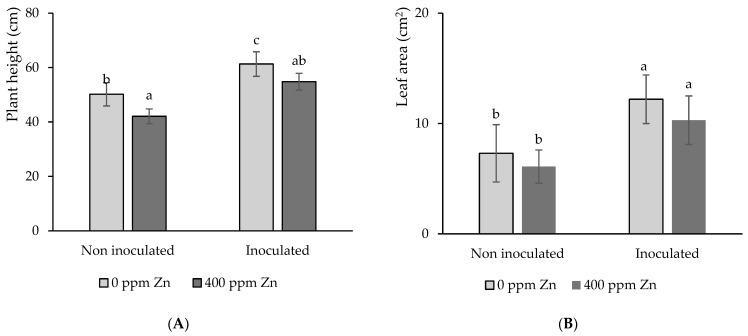
Impact of seed inoculation and 400 ppm Zn surplus on (**A**) plant height and (**B**) leaf area of tomato plants after 4 months’ growth. Values are means ± SD (n = 10). Different letters indicate differences between treatments at the 0.05 probability level.

Figure 5A,B shows the mean plant height and leaf area, respectively, of tomatoes at harvest time. Plant height was also affected by the addition of 400 ppm Zn to the substrate, showing a significant (*p* < 0.05) decrease of 16% relative to the control Zn-free treatment. Moreover, regardless of substrate Zn level, seed inoculation with biofilm had a beneficial impact on plant height.

However, plant height increases induced by inoculation were significantly (*p* < 0.05) different when comparing substrates with no Zn addition but not significant for substrates with Zn surplus. In the former case, inoculation led to a plant height increase of 30%, while, in the latter case, plant height increased 22% due to inoculation (Figure 5A). Therefore, the impacts of the Zn addition and inoculation in plant height were less important than those described above for shoot and root biomass.

With regard to leaf area, the Zn addition resulted in the development of smaller leaves relative to the control treatment. However, differences in leaf area were not significant (*p* < 0.05) between these two treatments, i.e., control and Zn. However, inoculation had a significant (*p* < 0.05) beneficial effect on leaf area, leading to increases of 67% and 69% in the presence and absence of Zn, respectively, compared to the non-inoculated counterparts (Figure 5B).

#### 3.4.2. Fruit Yield and Quality Evaluation

**Figure 6:**
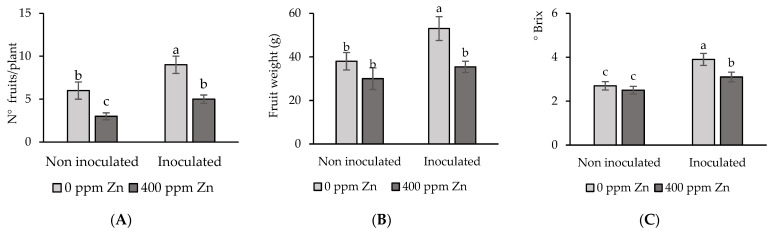
Impact of seed inoculation and 400 ppm Zn surplus on (**A**) fruit number; fruit weight (**B**) and quality index; Brix (**C**) of tomato plants after 4 months’ growth. Values are means ± SD (n = 10). Different letters indicate differences between treatments at the 0.05 probability level.

Tomato fruit is the edible and most important part of the plant. Hence, we evaluated fruit number, fruit weight, and fruit quality. The results of the four studied treatments are shown in Figure 6. Fruit number was strongly negatively impacted by Zn surplus, with a 50% reduction on average, i.e., considering inoculated and non-inoculated treatments, which was significant (*p* < 0.05) compared to Zn-free treatments. However, fruit number was enhanced by inoculation, showing significantly (*p* < 0.05) higher numbers in plants growing on both, substrates with or without metal (Figure 6A). Therefore, the positive impact of inoculation meant a 67% increase for the treatment with Zn surplus and a 50% increase for the treatment without Zn. Subsequently, under Zn stress, inoculation was found to alleviate metal toxicity, as the fruit number of the inoculated treatment with Zn surplus was of the same order of magnitude as that of the control treatment.

Zn surplus also negatively impacted fruit weight, as the treatment with extra metal exhibited a decrease of 21% with respect to the control treatment, even if this difference was not significantly (*p* < 0.05) different. However, inoculation increased fruit weight, which was 18% and 39% for plants growing on substrates with and without Zn addition, respectively, compared to the non-inoculated counterparts. The former differences were not significant (*p* < 0.05), while the latter showed significance at this probability level (Figure 6B).

The beneficial PGPB impacts have also been reflected in the soluble solids content (Brix), commonly used as a fruit quality index. Statistical results for Brix are similar to those obtained for fruit weight, with inoculation significantly (*p* < 0.05) enhancing the values of this parameter, irrespective of the substrate Zn level. Noteworthy, the Brix value of the inoculated treatment with no Zn addition (I) was higher than the control treatment, obtaining a value of 3.9, approaching the optimum Brix value of 4.0.

On average, tomato yields per plant for the studied treatments were 228 g (control), 477 g (I), 90 g (Zn), and 177 g (I + Zn). Adding 400 ppm Zn to the substrate very significantly (*p* < 0.001) decreased tomato yield (by 50% or more) compared to the control. This reduction was even more than 100% after comparing I and I + Zn treatments. Conversely, inoculation very significantly increased tomato yield, considering both the absence and excess Zn.

### 3.5. Elemental Composition in Response to Inoculation and Zinc Surplus

#### 3.5.1. Zn Accumulation in Root Shoot and Fruit

The concentrations of Zn in the control treatment were 34.10 ± 0.70 mg·kg^−1^ in root, 51.30 ± 10.40 mg·kg^−1^ in shoot, and 22.0 ± 2.60 mg·kg^−1^ in fruit. The rank of Zn levels as shoot > root > fruit found in the control treatment did not change in the treatment with seed inoculation (I). Zn concentration showed a trend to be higher with respect to the control in the inoculated metal-free treatment (I), and these differences were significant (*p* < 0.05) in the root but not in the shoot and fruit. In the absence of metal addition, seed inoculation, i.e., treatment I, triggered an increase of 25% of the Zn concentration in the tomato roots compared to the control treatment.

**Figure 7:**
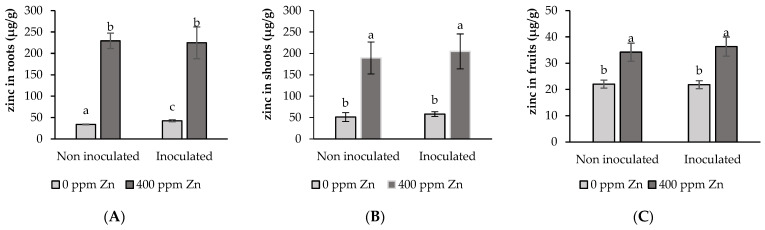
Impact of seed inoculation and 400 ppm Zn surplus on Zn levels at (**A**) root; (**B**) shoot; and (**C**) fruit of tomato plants after 4 months’ growth. Values are means ± SD (n = 10). Different letters indicate differences between treatments at the 0.05 probability level.

After four months of metal stress, the substrate addition of 400 ppm metal increased Zn concentrations by more than 600% in roots, 300% in shoots, and 40% in fruits, approximately, and this in both non-inoculated and inoculated treatments (Figure 7A–C). No significant differences (*p* < 0.05) in Zn concentration at root, shoot, and fruit (*p* < 0.05) were found between inoculated and non-inoculated treatments for substrates with a metal surplus. Opposite to the treatments without metal addition, in the treatments with Zn surplus in the substrate (Zn and I + Zn), Zn concentrations ranked as root > shoot > fruit.

#### 3.5.2. Concentration of Macro- and Microelements at Root, Shoot and Fruit

Table 2 and Table 3 show the concentrations of macro- and microelements in the root, shoot, and fruit of tomato for the studied treatments. Irrespective of seed inoculation or Zn stress, most elements showed a trend to accumulate in a specific part of the tomato plant. Thus, the microelements Fe, Cu, and Pb levels were clearly highest in root and ranked as root > shoot > fruit. The levels of the macroelements Ca, Mg, and S and the microelements Mn and Cd were highest in the shoot, ranking as shoot > root > fruit. Finally, P and K showed the highest concentrations in the fruit, ranking as fruit > shoot > root. Therefore, all the heavy metals analyzed in this work accumulate either in the root or in the shoot, showing the lowest concentrations in the fruit, the edible part of a tomato.

Translocation factors, TF_shoot_ = C_shoot_/C_root_ and TF_fruit_ = C_fruit_/C_root_, are listed in Tables S1 and S2 in Supplementary Materials. Within the macroelements, C, H, P, and K accumulate in the tomato fruit as the respective translocation factors, TF_frui_, were higher than 1. Nonetheless, TF_frui_ values were close to 1 for C and H and much higher for P and K. On the other hand, it is worth noting that all investigated heavy metals (Fe, Mn, Cu, Zn, Pb, and Cd) had translocation factors, C_fruit_/C_root_, lower than 1 (Table S2). This indicates that the investigated microelements did not accumulate in the edible part of the tomato plant. In other words, heavy metals accumulate in roots and shoots but not in the fruit of tomatoes.

Excess Zn in the substrate and seed inoculation showed no impact on the fruit concentrations of most of the elements studied in our work. The only exceptions were Cu and H. Treatments with Zn surplus (Zn and I + Zn) showed significantly (*p* < 0.05) higher Cu concentrations in the fruit than treatment I; however, they did not exhibit significant differences with the control treatment were found. Fruit H concentrations were significantly lower in the I and in the Zn treatments compared to the control treatment.

Similar to the fruit, in general, concentrations of most of the elements analyzed in shoot and root have not been significantly modified by the addition of Zn to the substrate, with only some exceptions. At the shoot, exceptions from this rule were found for C, N, and Cu, together with that of Zn before mentioned. Moreover, fruit C and N levels did not show significant differences. The shoot C concentrations of plants grown on substrates with 400 ppm Zn surplus were significantly (*p* < 0.05) lower compared to that of the control; however, these C concentrations showed no significant differences with the seed inoculated treatment without Zn surplus. Zn addition to the substrate resulted in decreased shoot N concentrations; however, this drop in N levels was only significant (*p* < 0.05) in the treatments with Zn plus seed inoculation and not significant when Zn was added, but inoculation was not performed. Contrasting with N results, Zn shoot concentrations increased in treatments with 400 ppm surplus; however, differences were significant only when seed inoculation in conjunction with Zn addition had been carried out.

The individual elements showing different concentrations between treatments at the root were the macronutrients K, Ca, and S as well as the micronutrient Mn, along with Zn, as it was anticipated. Root K concentrations have been found to be significantly (*p* < 0.05) higher in inoculated than in non-inoculated treatments, regardless of the Zn dose. The addition of Zn leads to higher root K concentrations only without inoculation. The addition of Zn to the substrate resulted in a significantly (*p* < 0.05) lower concentration of root Ca; on the contrary, the impact of seed inoculation on Ca root concentrations was significantly positive. Both the addition of Zn and inoculation leads to increased S root concentrations. However, root S levels were only significantly higher with respect to the control in the treatments combining excess Zn plus seed inoculation. Opposite to S, root concentrations of Mn showed a trend to decrease, both as a function of the Zn addition and seed inoculation. Again, levels of Mn were significantly (*p* < 0.05) lower with respect to the control for the treatment combining the Zn surplus and seed inoculation.

## 4. Discussion

### 4.1. Effects of Zinc on Bacterial Growth and Biofilm Production

In view of the above, ecofriendly strategies that can mitigate noxious Zn effects, are requested; among them, is the use of PGPRs. In particular, *B. subtilis* has been shown to be able to grow in cultures with excess Zn, even at potentially toxic metal concentrations. The prolongation of the initial latency or log phase of the *Bacillus* growth in Zn-enriched solutions (Figure 1) could indicate that the bacteria would have to adapt to metal excess. This initial adaptation could be driven by bacterial buffer systems allowing protection against toxic metals. These buffering systems would be operated by low molecular weight compounds that are able to hold together Zn, reducing its toxic effects. For example, bacillitliol (BSH), a low molecular weight compound, has been found to be a major component of the buffer system in *B. subtillis* [35]. Moreover, a longer log phase could be explained by the time required by the bacteria to activate other protection systems against heavy metals, such as the repression of metal uptake systems or the expression of proteins able to store the metal [35].

Heavy metal-tolerant bacteria have been shown to be efficient in synthesizing high amounts of exopolysaccharides, which also are mainly responsible for their resilience to excessive metal levels in soils or substrates [36,37,38]. Moreover, inoculant bacteria could develop metal tolerance by replacing metal-sensitive molecules with non-metal-sensitive ones. For instance, it has been reported that *B. subtilis*, growing under lethal concentrations of Zn, increased the production of a metal-insensitive cytochrome, namely, bd quinol oxidase, which would replace the conventional cytochrome oxidase [35].

Exopolysaccharide production and biofilm formation by soil microbial flora can be enhanced by the presence of a heavy metal itself, protecting the bacterium against toxicity [12]. In particular, it has been shown that the competitive advantages of *B. subtilis* in colonizing tomato roots are due to its ability to develop a biofilm [39]. Similarly, it has been suggested that root exudates of tomato plants would function as a trigger for biofilm formation, and several molecules in the rhizosphere could fulfill this function [40,41]. Accordingly, relationships between colonization ability, biofilm development, and biocontrol activity have been well established. This not only occurs in tomato plants but also in other plant species such as cucumber [42], cotton [43], banana [44], and Arabidopsis [40]. Accordingly, it was important to verify the ability of *B. subtilis* to produce biofilm when the studied metal was artificially added to the culture medium.

### 4.2. Effects of Zinc and Biofilm Inoculation on Seed Germination

Seed germination is a very sensitive stage for plant growth and can be affected by various external factors, including high levels of metals, which would be reflected in modifications of germination rate, root elongation, and/or germination rate. The addition of Zn to the substrate did not change the relative germination percentage (RG). However, the relative root elongation (RRE) was significantly increased (*p* < 0.05) at the 50 ppm Zn surplus, while it decreased between the 100 and 400 Zn surplus. These results could be linked to the phenomenon of Zn hormesis. This is a dose–response relationship, characterized by a beneficial impact to moderate stress and inhibiting reactions to the high pressure of an external factor, such as essential metals [45,46]. Therefore, the effect of Zn on RRE was consistent with its dual nature, as this element is essential at an optimum level and toxic at high levels. The germination index (GI) reflected toxic effects for the highest Zn concentrations tested, namely, 200 and 400 ppm. Thus, in the presence of such Zn levels, toxicity was ranked as high. However, seed inoculation with biofilm reduced toxicity from high to moderate for high Zn concentrations. Our results agree with previous reports about the impact of Zn on the RG and RRE [47] of *Coriandrum sativum* L. and *Medicago salitva* L. seeds. In contrast, *Cucumus sativusf* seeds exposed to increasing Zn concentrations showed increased inhibition of both germination and root elongation [48]. Overall, seed germination and seedling growth response to Zn stress would be specific to each plant species [49].

The positive effect of seed inoculation with biofilm on germination was reflected in an increase in RRE in inoculated seeds exposed to Zn in the range from 100 to 400 ppm. This effect could be due to the production of indole acetic acid (IAA) by *B. subtilis*, as it has been shown that this growth regulator induced increased root development in *Lactuca sativa* [18]. Moreover, a positive correlation between IAA production and root elongation induced by different *Bacillus* strains has been reported previously [50]. On the other hand, in the absence of seed inoculation, the germination index (GI) indicates toxic effects for the highest Zn concentrations tested, namely, 200 and 400 ppm. Thus, in the presence of such Zn levels, toxicity was ranked as high. However, seed inoculation with biofilm reduced toxicity from high to moderate for these high Zn concentrations.

### 4.3. Effects of Substrate Excess Zinc and Biofilm Inoculation on Plant Growth and Yield

The addition of excess Zn to the substrate resulted in significant reductions in tomato root and shoot biomass. Similar results have been found in 4-month-old tomato plants growing in a substrate with 250 ppm Zn [51]. Moreover, substrates enriched with ZnO nanoparticles, up to a Zn concentration of 400 ppm, produced decreased root and shoot tomato biomass [52]. The adverse impact of excess Zn on plant biomass has not only been reported for tomatoes, but also for coriander [47], rice, maize, *Triticum durum* [53], and *Triticum aestivum* [54]. A limited root development would restrict the volume of soil that the plant can explore for the uptake of water and nutrients, which would affect the growth of the entire plant. Seed inoculation with biofilm had a positive effect on both root and shoot growth. Noteworthy, plants growing from inoculated seeds on a Zn-contaminated substrate had a similar root and shoot biomass than those of the control treatment, which were growing from non-inoculated seeds and without substrate Zn surplus.

Similar to root and shoot biomass, substrate Zn excess impaired plant height and leaf area, while seed biofilm inoculation enhanced them. An excess of Zn has been shown to affect leaf area and leaf number in tomato plants. This has been attributed to the inhibition of either cell division, cell elongation, or both [51].

Negative impacts on tomato fruit yield and soluble solid content, assessed by the Brix index, also resulted from substrate Zn excess, while seed inoculation promoted positive trends of these parameters. Seed inoculation in the absence of Zn excess led to a Brix value close to 4, which corresponds to the reference value for fresh tomato consumption. Soluble solid concentration in tomato fruit has been demonstrated to be a major attribute for fresh fruits and processed products because it is directly related to sugar content, taste, aroma, and nutritional value [55]. Decreasing the yield of coriander plants exposed to high levels of Zn has been reported previously [47]. This was the result of reduced root and shoot biomass, which triggered a decline in nutrient uptake and a lower number of flowers and fruits.

Optimal Zn levels for biomass and fruit production again depend on plant species and may vary considerably. For example, negative effects on the growth of *Citrus reticulate* have been described at Zn concentrations of 650 ppm, while concentrations of 325 have been found to be considered optimal [56]. In our trials, however, concentrations as low as 100 ppm Zn have been demonstrated to be already harmful for tomato seed germination. In general, there is a narrow interval between Zn essentiality and toxicity in plants; therefore, this element plays a central role in agricultural sustainability.

Several mechanisms have been proposed to explain Zn potential noxious impacts on vegetables, and some examples are next enumerated. The overproduction of reactive oxygen species (ROS) has been shown to damage cell plants [57]. Decreased mitotic activity and irregularities at the chromosomal level have been proven to affect the vigor and yield of sugarcane plants [58]. A reduction in photoassimilates produced by leaves resulted in a decreasing biomass amount [59]. Modifications in root architecture, such as increased branching and the curvature of root areas in contact with the metal, inhibited cell proliferation and subsequently caused a reduction in the main root length [60], defoliation and retardation of leaf and root growth [56], etc.

Conversely, to cope with the harmful effects of Zn excess, plants have acquired a broad defense system. Root exudates are the first obstacle against heavy metal entry into the plant. This is because exudates are able to build up complexes with the metal, which reduces metal mobility. Other mechanisms limiting trace element access to the roots are expanded efflux of the metal from the cells and also biosorption in the cell walls [4,52]. Amino acids, phytochelatins, peroxide, glutathione, metallothioneins, and enzymes of the superoxide dismutase type are involved in the defense mechanisms [4].

Our results show that the inoculation of tomato seeds alleviated the negative impacts of a substrate contaminated with 400 ppm Zn on shoot and root biomass, fruit yield, and quality. Noteworthy, inoculation had no impact on the Zn concentrations of shoots, roots, and fruits. Therefore, the inoculated strain was able to boost added mechanisms to tackle the toxic effects of excess Zn. Moreover, the present work also allowed us to validate previous results, showing the efficiency of *B. subtilis* biofilm as an innovative method of inoculation of tomato seeds growing on an uncontaminated substrate [19]. Reasons explaining the efficiency of biofilm inoculation are next summarized.

First, the matrix structure of the biofilm, made of exopolysaccharides, allows itself to achieve intimate contact between seed and bacteria, providing greater opportunities for colonization. For example, it has been reported that biofilm application on tomato seeds was much more beneficial for plant growth than the conventional liquid inoculation in a planktonic state, using the same bacteria strain [19]. Moreover, biofilm exopolysaccharides have also been able to retain Zn after inoculation under conditions of metal surplus excess [9,12,13], thus reducing the amount of Zn in contact with the seed. Second, PGPR bacteria could favor plant growth in Zn-contaminated soils by changing the way in which the metal accumulates in the plant [61]. For example, *Brassica juncea* growing in a Zn-contaminated soil mainly accumulated the metal as oxalate or sulfate, while inoculation with *Rhizobium leguminosarum* resulted in a main accumulation of a Zn polygalacturonate complex. Moreover, when inoculated with *Pseudomonas brassicacearum*, this plant stored the metal mainly as Zn-cysteine [62]. Third, indeed, the beneficial effects of *B. subtilis* inoculation could be due to the various mechanisms enhancing the ability of the bacterium to act as a plant growth promotor and phytostimulant [18]. These tools may include nitrogen fixation [63], siderophore yielding [64], and phytohormone production, particularly indole acetic acid (IAA) and cytokinins, etc. [18]. All of these factors can modify cell morphogenesis and proliferation. This results in increased root development, allowing to increase in the volume of exploration in the soil for plant nutrition. Plant nutrition is also enhanced by PGPBs due to their ability to solubilize phosphorus [18,65,66]. What’s more, B. subtilis has been found to survive inside the root of tomato plants, indicating that its effects could persist throughout the successive stages of the entire crop growth season [19]. Finally, symbiotic relationships may develop between plants and various microorganisms due to stress effects caused by heavy metals. As a result, valuable effects can be induced on plants by direct or indirect mechanisms, such as, for example, the production of biofilm, phytohormones, exopolysaccharides, and/or siderophores. Thus, symbiotic associations would be advantageous, as they are able to upgrade metal tolerance and increase biomass production [4]. Conversely, microbial populations in soil can also be severely affected by excess Zn. For example, Zn resulted in negative effects on biological N fixation by *Rhizobia* with a subsequent decrease in plant biomass [67]. In this way, the use as inoculants of PGPB microorganisms that are resistant to metal could, in part, counteract the negative effect of metal stress.

Overall, our results suggest that the growth-promoting effect triggered by *B. subtilis* on tomato plants cropped on substrates with extra Zn was not associated with the retention of the metal, i.e., the prevention of its ingress into the plant. In fact, seed inoculation without extra Zn (treatment I), significantly increased the concentration of this metal in roots (Table 3). Thus, as indicated above, the advantage of using *B. subtilis* would be associated with plant growth-promoting effects induced by the different tools available by this inoculant (such as biological N fixation, P solubilization, and the release of auxin and cytokinin-type growth regulators, etc.), which benefit plant development as a whole. Regardless, this work shows that seed inoculation with biofilm achieved its plant growth-promoting effect on tomatoes, even under the stress conditions imposed by the high Zn levels.

### 4.4. Effects of Substrate Excess Zinc and Biofilm Inoculation on Elemental Composition

Irrespective of Zn level and inoculation treatment, concentrations of Zn in the tomato Río Grande variety were much lower in fruits than in shoots and roots. Fruit Zn concentrations ranged from 21.80 ± 1.80 mg kg^−1^ to 36.30 ± 3.60 mgkg^−1^, and this was in spite of significant (*p* < 0.05) differences between excess Zn versus control with no added Zn treatments. Tomato plants growing in substrates with extra Zn showed much higher levels of this metal in roots, shoots, and fruits than those growing in substrates without a Zn addition, and this is either with or without seed inoculation. The former exhibited higher Zn levels than the latter by factors of about 5.7 to 7 for roots, 3 to 3.5 for shoots, and 1.5 to 1.6 for fruits.

The zinc levels of tomato fruits growing on soils and substrates have been reported by several authors [24,25,68,69] and are summarized in Table S3 as Supplementary Materials. The factors studied included soil contamination, fertilization and substrate depth, cultivar type, etc. Previously reported tomato fruit Cu concentrations are highly variable. For example, in a field experiment involving contaminated and non-contaminated farms, reported values ranged from 5.17 to 59.67 mg/Kg Zn [25]. However, higher values ranging from 132.27 to 192.27 mg/Kg Zn in a greenhouse experiment analyzing the effect of CO_2_ concentration on two different tomato varieties have also been revealed [24]. Therefore, the Zn concentrations for tomato fruits obtained in this work fit within the wide range of values previously made known.

A much higher accumulation of Zn and other heavy metals in roots compared to other plant organs has been widely reported [70,71]. Root Zn stockpiling has been viewed as a plant strategy to keep Zn concentrations low in edible parts [8]. This scheme is particularly important for a vegetable such as tomato that produces fruits fit to be eaten, while neither roots nor leaves are consumed as food. Our results are consistent with the idea that in several plant species, roots can gather metal contamination and prevent translocation to fruits or in general to plant edible parts. In other words, root Zn stockpiling has been viewed as a plant strategy to keep metal concentrations low in edible parts [8]. This scheme is particularly important for vegetables such as tomatoes that produce fruits fit to be eaten, while neither roots nor leaves are consumed as food. On top of that, it has been reported that, in tomatoes, Zn mainly accumulates in the root cortex and also in the secondary veins of leaves, keeping its concentration comparatively very low in fruits [72]. The accumulation of Zn in the cell walls of roots and leaves has also been proposed as one of the mechanisms of plant protection against toxic metals, moving them away from metal-sensitive sites [73].

Root, shoot, and fruit Zn concentrations of plants growing from inoculated seed on a substrate with 400 ppm excess metal (I + Zn) showed no differences compared to the noninoculated counterpart with excess Zn (Zn). Moreover, the seed inoculation of plants growing on a substrate without added metal significantly (I) (*p* < 0.1) enhanced root Zn level, but did not change shoot and fruit Zn levels with respect to the control treatment. Thus, inoculation had no effects on Zn concentrations of root, shoot, or fruit Zn in any of the treatments investigated, except for root Zn levels in the I treatment, which were higher (42.60 ± 2.40 mg kg^−1^), relative to the control (34.10 ± 0.70 mg kg^−1^). This is an unexpected result as it has been found that Zn adsorption on the biofilm components rather could decrease the concentration of this element in direct contact with the roots, thus triggering the protective effect of the inoculant. Indeed, it has been demonstrated that *B. subtilis* associates or adheres to the root surface developing a biofilm [74,75]. Moreover, it has been suggested that Zn adsorption to biofilm results in a protective mechanism for the bacteria, allowing tolerance of toxic metal concentrations [36,76]. Moreover, several authors have reported that various bacteria with PGPR attributes are able to affect the mobility and availability of heavy metals through different mechanisms such as biosorption, intracellular accumulation, precipitation, redox processes, and methylation, among others [77,78,79,80]. Therefore, a decline, but not an increase in Zn concentration, mainly in the roots, was expected after seed inoculation.

In general, macro- and microelement concentrations in tomato roots, shoots, and fruits showed no significant differences between the seed inoculation and substrate Zn level treatments studied. Exceptions were Cu and H in the fruit, C, N, and Cu in the shoot, and, finally, K, Ca, S, and Mn in the root, which showed significant differences at the *p* < 0.1 level. Seed inoculation showed trends to either decrease or increase both Cu and H fruit concentrations, without and with extra Zn in the substrate, respectively. Substrate Zn addition showed a trend to increase fruit Cu concentrations. These trends, however, were not always significant.

Carbon and nitrogen levels in the shoot were higher for the control treatment than for the rest of the treatments. Higher C concentrations have been found for the control treatment, relative to the rest of the treatments, which were significant (*p* < 0.01) for Zn and Zn + I treatments. Concentrations of N were significantly higher in the control treatment compared to the I + Zn treatment. Decreasing N concentrations with increasing substrate Zn have also been observed in other crops such as maize and citrus [81]. This trend could be related to the observed decrease in root elongation and root weight, as N is the most essential element for crop growth [82]. Extra Zn in the substrate significantly (*p* < 0.01) increased Cu shoot concentrations, while inoculation showed a non-significant trend to decrease shoot Cu levels.

Inoculation had a positive and significant effect on root S and K concentrations, both with and without Zn surplus in the substrate. It has been reported that the permeases of plant-associated microorganisms play a crucial role in the sulfate uptake by plant roots [83]. With regard to K, despite being a very common element, it is mostly bound to soil/substrate compounds, and, therefore, it needs to be added as a fertilizer to be available for plants. The K increase in the roots of inoculated plants could be explained by the solubilization mechanism previously reported for *Bacillus* [84]. In addition, it has been demonstrated that inoculation with a potassium-solubilizing bacterium, *Enterobacter hormaechei*, increases the growth of tomato plants [85]. The inoculation of tomato seeds with biofilm induced an increase in S in the roots, both in the absence and presence of zinc. However, significant differences (*p* < 0.01) have only been evidenced when the I + Zn and the control treatments were compared. This effect could be explained by the ability of *Bacillus* to act on the sulfur cycle, which increases tomato production [85,86]. Extra Zn in the substrate also significantly decreased root Ca concentration of both inoculated and non-inoculated plants. It has been argued [8] that high zinc concentrations interfere with nutrient uptake, including Ca. Inoculation, however, increased root Ca concentration, and this rise was more important for plants growing on a metal-free substrate. A previous study reported root Ca increases due to inoculation by *Bacillus* sp. LJ16 strain in tomato [23]. Within the heavy metals, only Mn root concentrations showed a trend to decrease both as a function of Zn surplus and inoculation. Significant differences (*p* < 0.10) have only been found between the control and I + Zn treatments.

Alterations in the levels of several other elements have been reported following high Zn concentrations in soils/substrates. Excess Zn has been demonstrated not only to interfere with the uptake of nutrients (P, N, K, Ca, Mg, Fe…) by roots but also to affect their allocation to different plant organs [8,68,87]. These results, however, have not been corroborated in our work. The intake of nutrients from the substrate and their distribution in roots, shoots, and fruits has been shown to depend on plant species and plant organs [8,68].

### 4.5. Implications and Future Perspectives

The scope of this study was not to assess the risk of Zn accumulation in tomatoes for human health. However, a rough evaluation of tomato safety regarding Zn levels obtained in tomato fruit has been performed, extrapolating data from previous works [25,68,69] and simple calculations. Therefore, assuming a mean day consumption of 200 mg tomato for an adult, no safety concerns are posed when using the tomato fruits with the maximum concentrations of Zn measured, which were obtained in the contaminated substrate.

High levels of Zn in soils result from man-made additions and weathering. Zinc contents are higher in soils derived from basic eruptive rocks than in other soils [7]. Moreover, Zn contamination has been found to be more important in sludge-amended soils [8,30,68] but may be induced by other anthropic inputs, such as, for example, irrigation with contaminated water [25]. Zinc toxicity in plants is far less widespread than Zn deficiency; however, as stated, it may be prevalent worldwide in horticultural belts that have been developed in the vicinity of many large urban centers in soils [7,30]. In this area, PGBPs, together with other microbial and biotechnological approaches, are thought to be able to mitigate the negative impact of Zn in vegetable growth and the potential negative effects on the heavy metal content of the edible parts of crops.

Various inoculant products currently on the market for promoting plant growth have been obtained using *B. subtilis*, and the cost of these products is affordable [14]. Moreover, it is important to establish the appropriate inoculation method, as it directly affects PGPB survival and colonization. Previous work [17,18,19] has shown the efficiency of using biofilm from *B. subtilis* compared to other methods. To produce biofilm, glycerol was used as a carbon source. Glycerol was obtained as a sub-product from biodiesel synthesis and, therefore, it is a byproduct, reducing manufacturing costs.

Most PGPB research on alleviation environmental stress of Zn and other metals has been carried out in pot or greenhouse experiments. Moreover, PGPBs are easily affected by environmental factors, namely, temperature, soil pH, redox conditions, and so on, which may hamper the bioremediation efficiency. Therefore, field research is needed to better understand the interaction between major factors, namely, metal, soil, microorganisms, and plants. Due to the high specificity between plants and beneficial microorganisms, it would be of interest to evaluate whether this new method of seed bioinoculation, through the application of biofilm, could protect other tomato varieties from the toxic effects of zinc. Moreover, there is a need to optimize practical and easily applicable strategies in different regions, crops, and soils worldwide. Therefore, future research also needs to focus on the evaluation of different approaches for different man-made contamination sources with the aim of achieving more effective results.

## 5. Conclusions

The inoculation of tomato seeds with biofilm obtained from *B. subtilis* subsp. *spizizenii* boosted first germination and then plant growth and production, even under stress conditions imposed by excess Zn in the substrate. Therefore, biofilm inoculation has been validated as a tool to support the environmental sustainability of tomato plants growing in greenhouse conditions. Moving PGPB technology from the laboratory to the field could facilitate sustainable crop growth and reduction in fertilizer and pesticide use in Zn-contaminated arable land.

Overall, our results suggest that the beneficial effects triggered by *B. subtilis* subsp. *spizizenii* on tomato plants, growing in substrates with extra Zn, were not linked to mechanisms associated with the external retention of the metal, preventing its entry into the root. Rather, tomato growth improvements by seed inoculation could be related to widely known mechanisms associated with plant growth-promoting effects triggered by PGPBs, such as, for example, biological N fixation, P solubilization, the release of auxin and cytokinin-type growth regulators, etc., which contribute to globally increasing plant production.

The investigated heavy metals (Fe, Mn, Cu, Zn, Pb, Cd) did not accumulate in the tomato fruit, as translocation factors C_fruit_/C_root_ were lower than 1. Within the macroelements, P and K accumulation on tomato fruit showed high rates. The addition of extra Zn to the substrate did not affect the concentrations of fruit microelements other than Zn itself; moreover, seed inoculation had no impact on any fruit microelement concentrations, including Zn.

## Figures and Tables

**Table 1:** Phytotoxicity criteria that were established according to [28] from relative germination index.

Germination Index (GI%)	Toxicity Level
<50%	High
50–80%	Moderate
>80%	No toxicity

**Table 2:** Macroelement concentration on tomato root, shoot, and fruit in response to inoculation and Zn surplus. Treatments are controlled, non-inoculated seeds growing without added Zn; I, inoculated seeds growing without added Zn; Zn, non-inoculated seeds growing with Zn surplus of 400 ppm; I + Zn, inoculated seeds growing with Zn surplus of 400 ppm. Values are means ± SD (n = 3). Different letters indicate significant differences between treatments at the *p* < 0.10 probability level for root (lower case letters), shoot (upper case letters), and fruit (italics and lower case) independently.

		Percent Carbon	Percent Hydrogen	Percent Nitrogen	Sulfur (mg/g)	Phosphorus (mg/g)	Potassium (mg/g)	Calcium (mg/g)	Magnesium (mg/g)
Root	Control	38.01 ± 2.88 a	5.37 ± 0.26 a	1.91 ± 0.12 a	4.79 ± 0.40 a	2.21 ± 0.45 a	11.22 ± 1.39 a	13.31 ± 1.29 b	2.99 ± 0.56 a
	I	38.42 ± 2.21 a	5.33 ± 0.38 a	2.01 ± 0.15 a	6.49 ± 0.79 a,b	2.54 ± 0.26 a	20.73 ± 4.61 b,c	18.93 ± 0.35 c	3.09 ± 0.1 a
	Zinc	37.25 ± 2.68 a	4.92 ± 0.2 a	1.87 ± 0.18 a	5.08 ± 2.78 a,b	2.68 ± 0.42 a	17.62 ± 3.16 b	11.88 ± 0.65 a	3.37 ± 1.06 a
	I + Zinc	37.54 ± 2.95 a	5.11 ± 0.27 a	1.85 ± 0.26 a	7.62 ± 1.62 b	2.93 ± 0.52 a	25.08 ± 5.33 c	14.56 ± 1.14 b	3.55 ± 0.95 a
Shoot	Control	35.09 ± 0.46 B	5.27 ± 0.14 A	2.02 ± 0.05 B	20.83 ± 2.02 A	3.47 ± 0.55 A	28.77 ± 4.24 A	34.46 ± 1.65A	7.05 ± 0.28 A
	I	34.12 ± 0.45 A,B	5.09 ± 0.1 A	1.94 ± 0.12 B	24.58 ± 5.3 A	2.8 ± 0.38 A	32.92 ± 5.7 A	34.9 ± 7.92 A	8.17 ± 1.01 A
	Zinc	33.26 ± 1.12 A	5.15 ± 0.12 A	1.67 ± 0.41 A,B	20.16 ± 4.04 A	4.03 ± 2.2 A	35.44 ± 5.71 A	35.3 ± 8.93 A	8.19 ± 1.42 A
	I + Zinc	33.85 ± 0.51 A	5.1 ± 0.11 A	1.37 ± 0.36 A	25.04 ± 1.18 A	2.64 ± 0.24 A	32.23 ± 4.25 A	39.77 ± 4.03A	7.48 ± 0.55 A
Fruit	Control	40.24 ± 0.55 a	5.94 ± 0.25 c	1.77 ± 0.43 a	1.9 ± 0.37 *a*	5.14 ± 0.54 *a*	46.96 ± 2.67 *a*	0.79 ± 0.19 *a*	1.54 ± 0.32 *a*
	I	41.34 ± 0.11 a	5.53 ± 0.07 a	1.97 ± 0.3 a	1.93 ± 0.21 *a*	4.92 ± 0.84 *a*	49.88 ± 3.05 *a*	0.93 ± 0.09 *a*	1.31 ± 0.07 *a*
	Zinc	41.26 ± 0.79 a	5.64 ± 0.05 a,b	1.71 ± 0.37 a	1.81 ± 0.34 *a*	4.93 ± 0.23 *a*	47.32 ± 2.08 *a*	0.83 ± 0.03 *a*	1.37 ± 0.09 *a*
	I + Zinc	41.78 ± 0.61 a	5.76 ± 0.1 b,c	1.72 ± 0.44 a	2.13 ± 0.34 *a*	5.43 ± 0.71 *a*	51.41 ± 6.56 *a*	0.95 ± 0.12 *a*	1.59 ± 0.21 *a*

**Table 3:** Microelements concentration on tomato root, shoot, and fruit in response to inoculation and Zn surplus. Treatments are controlled, non-inoculated seeds growing without added Zn; I, inoculated seeds growing without added Zn; Zn, non-inoculated seeds growing with Zn surplus of 400 ppm; I + Zn, inoculated seeds growing with Zn surplus of 400 ppm. Values are means ± SD (n = 3). Different letters indicate significant differences between treatments at the *p* < 0.10 probability level for root (lower case letters), shoot (upper case letters), and fruit (italics and lower case) independently.

		Iron (µg/g)	Manganese (µg/g)	Cooper (µg/g)	Zinc (µg/g)	Lead (µg/g)	Cadmium (µg/g)
Root	Control	1615.67 ± 696.44 a	52.03 ± 12.82 b	14.99 ± 1.4 a	34.10 ± 0.70 a	14.19 ± 7.86 a	0.25 ± 0.04 a
	I	1026.75 ± 727.5 a	39.08 ± 12.49 a,b	12.42 ± 2.01 a	42.60 ± 2.40 b	8.05 ± 1.84 a	0.24 ± 0.03 a
	Zn	1235.88 ± 669.59 a	43.54 ± 14.56 a,b	11.57 ± 1.61 a	229.40 ± 18.00c	11.58 ± 10.26 a	0.18 ± 0.07 a
	I + Zn	786.33 ± 157.12 a	29.51 ± 7.05 a	13.6 ± 3.55 a	224.70 ± 37.20c	6.44 ± 3.58 a	0.19 ± 0.05 a
Shoot	Control	417.85 ± 170.7 A	74.45 ± 26.61 A	8.06 ± 2.49 A	51.30 ± 10.40 a	2.51 ± 1.27 A	0.31 ± 0.11 A
	I	507.88 ± 118.01 A	55.24 ± 3.81 A	8.01 ± 2.11 A	58.30 ± 5.50 a	3.69 ± 0.77 A	0.42 ± 0.06 A
	Zn	394.43 ± 119.3 A	72.72 ± 12.87 A	11.96 ± 2.55 B	189.40 ± 37.40 b	2.97 ± 0.082 A	0.35 ± 0.12 A
	I + Zn	381.26 ± 17.68 A	78.18 ± 0.90 A	9.57 ± 1.83 A,B	204.80 ± 40.70 b	2.64 ± 0.08 A	0.36 ± 0.02 A
Fruit	Control	33.46 ± 12.73 *a*	8.56 ± 2.42 *a*	7.64 ± 1.29 *a,b*	22.00 ± 2.60 a	0.4 ± 0.08 *a*	<0.06
	I	42.42 ± 14.44 *a*	6.84 ± 0.82 *a*	5.11 ± 1.00 *a*	21.80 ± 1.80 a	0.38 ± 0.12 *a*	<0.06-
	Zn	49.11 ± 19.77 *a*	8.02 ± 0.91 *a*	9.68 ± 1.60 *b*	34.20 ± 2,90 b	0.17 ± 0.01 *a*	-<0.06
	I + Zn	40.06 ± 5.93 *a*	9.6 ± 2.31 *a*	10.88 ± 5.01 *b*	36.30 ± 3.60 b	0.39 ± 0.27 *a*	<0.06-

## Data Availability

The original contributions presented in the study are included in the article/Supplementary Materials, further inquiries can be directed to the corresponding author.

## Supplementary Materials

The following supporting information can be downloaded at: https://www.mdpi.com/article/10.3390/microorganisms12112237/s1, Figure S1. Biofilm produced by *B. subtilis* at the air–liquid interface. (Culture medium was a minimum salt medium with 1% glycerol 35mM glutamic acid). Figure S2. Biofilm adhered to the walls of an Erlenmeyer flask, after removing the liquid medium. Table S1. Mean translocation factors, C_shoot_/C_root_ and C_shoot_/C_Root_ for macroelements: C, H, N, P, K, Ca, Mg, and S; Table S2. Mean translocation factors, C_shoot_/C_root_ and C_shoot_/C_root_ for microelements: Fe, Mn, Cu, Zn, Pb, and Cd; Table S3. Maximum and minimum concentrations of Zn in tomato fruits reported in previous works.
