# Supplementary material for: The Effects of Seed Inoculation with Bacterial Biofilm on the Growth and Elemental Composition of Tomato (Solanum lycopersicum L.) Cultivated on a Zinc-Contaminated Substrate
Source: Microorganisms. 2024 Nov 5;12(11):2237. doi: 10.3390/microorganisms12112237 (PMC11596727; doi:10.3390/microorganisms12112237)
Supplement: Supplementary file 1 [file microorganisms-12-02237-s001.zip › microorganisms-3261968-supplementary.pdf]

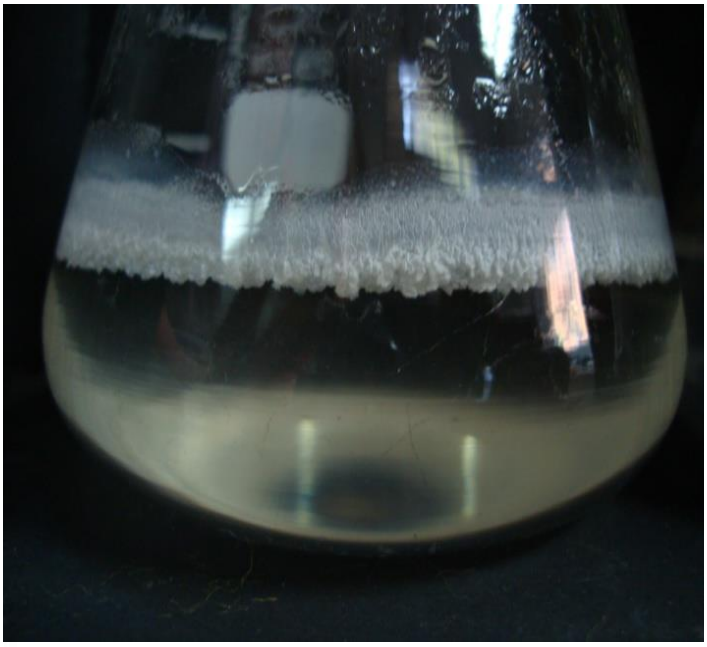

Figure S1. Biofilm produced by *B. subtilis* at the air-liquid interface.
(Culture medium was a Minimum Salt Medium with
1% glycerol 35mM glutamic acid).

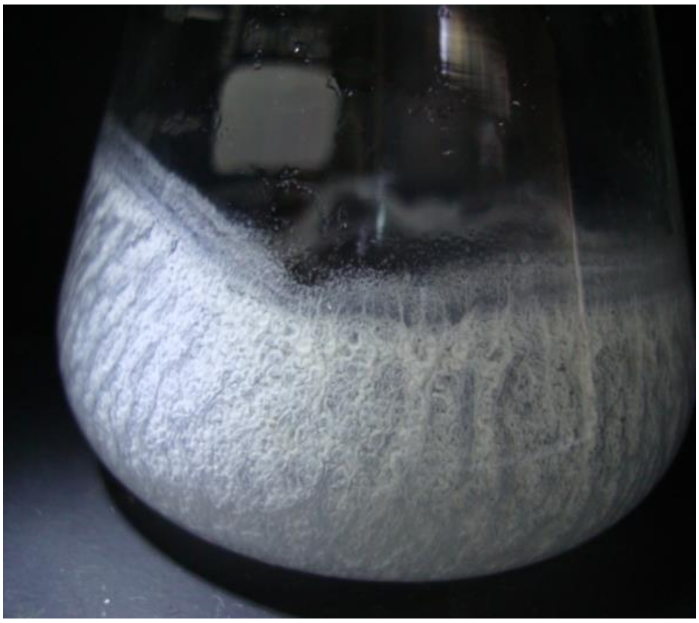

Figure S2. Biofilm adhered to the walls of an Erlenmeyer flask,
after removing the liquid medium

Table S1. Mean translocation factors, C_{shoot}/C_{root} and C_{fruit}/C_{root} for macroelements: C, H, N, P. K, S Ca and Mg.

		C (%)	H (%)	N(%)	S (mg/g)	P (mg/g)	K (mg/g)	Ca (mg/g)	Mg (mg/g)
Shoot/Root	Control	0.93	0.98	1.06	3.25	1.39	1.42	2.60	2.28
	I	0.89	0.96	0.97	5.19	1.31	2.97	1.84	2.76
	Zn	0.90	1.05	0.90	5.03	1.50	2.09	2.42	2.54
	I+Zn	0.90	1.00	0.73	3.37	0.91	1.36	3.37	2.23
Fruit/Root	Control	1.06	1.11	0.94	0.30	2.03	2.34	0.06	0.50
	I	1.08	1.04	0.99	0.41	2.31	4.50	0.05	0.45
	Zn	1.11	1.15	0.91	0.55	1.89	2.74	0.06	0.45
	I+Zn	1.12	1.13	0.93	0.29	1.89	2.10	0.08	0.46

Table S2. Mean translocation factors, C_{shoot}/C_{root} and C_{fruit}/C_{root} for microelements: Fe, Mn, Cu, Zn, Pb and Cd

		Fe (μg/g)	Mn (μg/g)	Cu (μg/g)	Zn (μg/g)	Pb (μg/g)	Cd (μg/g)
Soot/Root	Control	0.56	2.03	0.67	1.51	0.32	1.34
	I	0.36	1.12	0.53	1.37	0.30	1.69
	Zn	0.36	1.79	1.04	0.84	0.56	2.18
	I+Zn	0.50	2.76	0.72	0.94	0.52	1.98
Fruit/Root	Control	0.04	0.22	0.62	0.64	0.04	-
	I	0.03	0.14	0.35	0.51	0.02	-
	Zn	0.06	0.20	0.85	0.15	0.03	-
	I+Zn	0.05	0.35	0.83	0.17	0.05	-

Table S3. Maximum and minimum concentrations of Zn in tomato fruits reported in previous works.

Factor studied	Maximum Zn (g. kg^{-1})	Minimum Zn (g. kg^{-1})	Reference
Tomato variety	196.27	132.27	Khan et al., 2013
Temperature regime, variety	14.10	26.50	Pimenta et al., 2022
Substrate depth and fertilizer	8.10	10.92	Nektarios et al., 2022
Soil contamination	59.67	5.17	Ahmed et al., 2023
